# From array-based hybridization of *Helicobacter pylori *isolates to the complete genome sequence of an isolate associated with MALT lymphoma

**DOI:** 10.1186/1471-2164-11-368

**Published:** 2010-06-10

**Authors:** Jean-Michel Thiberge, Caroline Boursaux-Eude, Philippe Lehours, Marie-Agnès Dillies, Sophie Creno, Jean-Yves Coppée, Zoé Rouy, Aurélie Lajus, Laurence Ma, Christophe Burucoa, Anne Ruskoné-Foumestraux, Anne Courillon-Mallet, Hilde De Reuse, Ivo Gomperts Boneca, Dominique Lamarque, Francis Mégraud, Jean-Charles Delchier, Claudine Médigue, Christiane Bouchier, Agnès Labigne, Josette Raymond

**Affiliations:** 1 Institut Pasteur, Génotypage des Pathogènes et Santé Publique, Paris, France; 2 Institut Pasteur, PF4 Analyse et Intégration Génomiques, Paris, France; 3 Université Victor Segalen, INSERM U853, Bordeaux, France; 4 Institut Pasteur, PF2 Puces à ADN, Paris, France; 5 Institut Pasteur, PF1 Génomique, Paris, France; 6 CEA, Direction des Sciences du Vivant, Institut de Génomique, Genoscope & CNRS-UMR 8030, Laboratoire d'Analyse Bioinformatique en Génomique et Métabolisme, Evry, France; 7 CHU de Poitiers, EA4331, LITEC, Bactériologie, Poitiers, France; 8 Hôpital Saint Antoine, Paris, France; 9 Hôpital Villeneuve Saint Georges, France; 10 Institut Pasteur, Unité postulante de Pathogenèse de Helicobacter, Paris, France; 11 Institut Pasteur, Groupe Biologie et génétique de la paroi bactérienne, Paris, France; 12 INSERM, Groupe Avenir, Paris, France; 13 Hôpital Hôtel Dieu, Paris, France; 14 Hôpital Henri Mondor, Créteil, France; 15 Université Paris Descartes, Faculté de Médecine, Hôpital Cochin, Paris, France

## Abstract

**Background:**

*elicobacter pylori *infection is associated with several gastro-duodenal inflammatory diseases of various levels of severity. To determine whether certain combinations of genetic markers can be used to predict the clinical source of the infection, we analyzed well documented and geographically homogenous clinical isolates using a comparative genomics approach.

**Results:**

A set of 254 *H. pylori *genes was used to perform array-based comparative genomic hybridization among 120 French *H. pylori *strains associated with chronic gastritis (n = 33), duodenal ulcers (n = 27), intestinal metaplasia (n = 17) or gastric extra-nodal marginal zone B-cell MALT lymphoma (n = 43). Hierarchical cluster analyses of the DNA hybridization values allowed us to identify a homogeneous subpopulation of strains that clustered exclusively with *cag*PAI minus MALT lymphoma isolates. The genome sequence of B38, a representative of this MALT lymphoma strain-cluster, was completed, fully annotated, and compared with the six previously released *H. pylori *genomes (i.e. J99, 26695, HPAG1, P12, G27 and Shi470). B38 has the smallest *H. pylori *genome described thus far (1,576,758 base pairs containing 1,528 CDSs); it contains the *vacA*s2m2 allele and lacks the genes encoding the major virulence factors (absence of *cag*PAI, *bab*B, *bab*C, *sab*B, and *hom*B). Comparative genomics led to the identification of very few sequences that are unique to the B38 strain (9 intact CDSs and 7 pseudogenes). Pair-wise genomic synteny comparisons between B38 and the 6 *H. pylori *sequenced genomes revealed an almost complete co-linearity, never seen before between the genomes of strain Shi470 (a Peruvian isolate) and B38.

**Conclusion:**

These isolates are deprived of the main *H. pylori *virulence factors characterized previously, but are nonetheless associated with gastric neoplasia.

## Background

*Helicobacter pylori *infections occur in approximately 50% of the human population and are associated with several inflammatory gastroduodenal diseases [1], including two types of gastric cancers: gastric adenocarcinoma [2] and gastric extra-nodal marginal zone B-cell MALT (mucosa-associated lymphoid tissue) lymphoma, first described by Isaacson et al. [3]. Evolution of this bacterial infection towards malignancy only occurs in approximately 1% of infected individuals, suggesting that both bacterial and host susceptibility factors are involved[4].

Since the discovery of *H. pylori*, several studies have focused on elucidating *H. pylori *pathogenicity mechanisms (microbial factors) that are associated with disease outcomes[5]. The *cag*-pathogenicity island (*cag*PAI) has been recognized as a major pro-inflammatory actor, but its association with MALT lymphoma strains has yet to be clearly shown [6]. The VacA vacuolating cytotoxin, thought to cause detectable alterations in gastric epithelial cells and immune cells, is also one of the most studied *H. pylori *virulence factors [7]. VacA has also been suggested to play a role in *H. pylori *persistence, demonstrated by *in vitro *studies, based on its immunosuppressive properties [8]. Adhesion of *H. pylori *to gastric epithelial cells is another bacterial trait contributing to chronic state of the infection. BabA [9], SabA [10], HopZ [11], HomB [12] and 30 outer-membrane-like paralogs recognized as adhesins or potential adhesins are encoded by the *H. pylori *genome [13]. Several studies have highlighted their contribution to pathogen fitness in human populations [14,15]. Over the last twenty years, genes encoding these virulence factors have served as genotyping markers to establish correlations between these markers, alone or in combination, and clinical outcomes of *H. pylori *infections [16].

Few studies have been conducted in relation to gastric MALT lymphoma-associated strains. Koehler *et al. *reported that the *vacA*m2 allele predominated in MALT lymphoma-associated isolates [17]. In previous studies [18,19] including an identical collection of *H. pylori *gastric MALT lymphoma strains to that used here, the authors confirmed this finding and suggested that certain combinations of genomic markers may have a predictive value for determining whether gastric MALT lymphoma develops. All these data suggest the potential role for bacterial determinism in the clinical outcome of MALT lymphoma.

So far, comparative genomics involving sequenced *H. pylori *genomes have been limited to five clinical isolates isolated in the West and associated with gastritis [strain 26695 [20], peptic ulcers (strains J99 [GenBank:AE001439.1], P12 [EMBL:CP001217, EMBL:CP001218]), atrophic gastritis (HPAG1 [21]), or no known disease (strains G27 [22] and Shi470 [RefSeq:NC_010698]. However, no genome sequence of a *H. pylori *strain isolated from MALT lymphoma is currently available. Comparative genomics based on DNA-array analyses, first conducted by Salama *et al. *on 15 Caucasian isolates [23], led to the elucidation of the *H. pylori *core genome comprising the pool of ubiquitous *H. pylori *genes and strain-specific genes (non-ubiquitous). Gressmann *et al. *studied gene gain and loss during evolution, by comparing the genome of 56 globally representative strains of *H. pylori*; they reported that 25% of the genes were non-ubiquitous [24]. Through comparative genomics based on the analysis of 24 clinical isolates from various geographical origins (Western, Asian, African countries) using whole genome DNA arrays, we identified 213 non-ubiquitous or strain-specific genes [25]. In this study, we describe the gene distribution of these 213 non-ubiquitous genes (Additional file 1) within genomes from a large geographically homogeneous French collection of 120 well-characterized *H. pylori *strains associated with chronic gastritis, duodenal ulcer, intestinal metaplasia or gastric MALT lymphoma. A hierarchical clustering analysis of the DNA hybridization values identified a homogeneous phylogenic subpopulation of strains containing all of the *cag*PAI minus MALT lymphoma isolates. The B38 isolate was selected as a representative of this MALT lymphoma-specific cluster. Its genome sequence was completed, fully annotated, and compared with previously sequenced and published *H. pylori *genomes.

## Results and Discussion

### Non-ubiquitous gene distribution in relation to associated diseases

**Figure 1:**
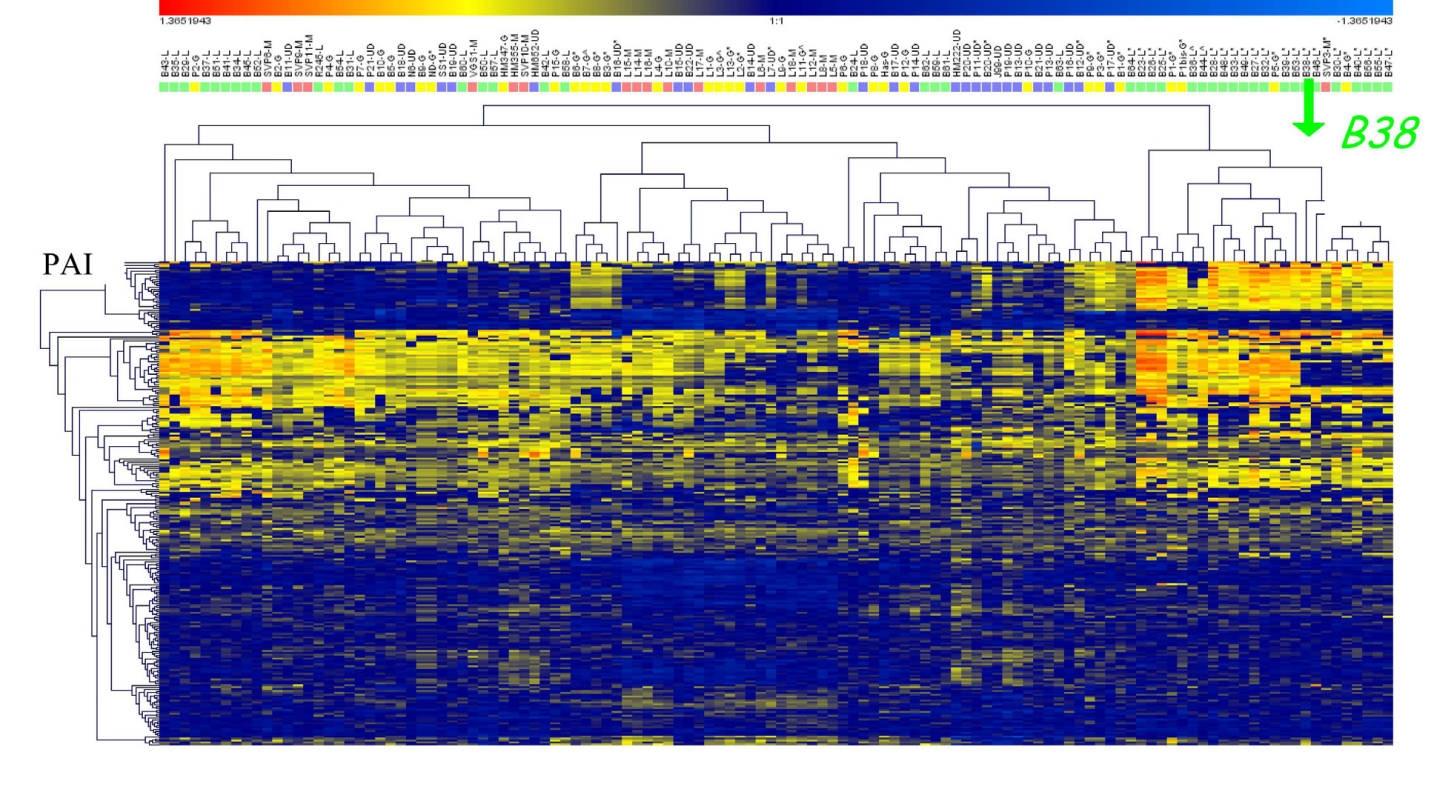
**Hybridization reactions on a DNA macroarray membrane containing 254 PCR products that are representative of *H. pylori *strain 26695 (41 ubiquitous genes + 213 non-ubiquitous or strain-specific genes)**. Bacterial DNAs from 120 isolates involved in various diseases, including chronic gastritis (yellow), intestinal metaplasia (pink), duodenal ulcer (blue) and gastric MZBL (green), were tested by hybridization. Isolates are listed on the horizontal axis, and the genes tested, on the vertical axis. Clustering (genesis software) was carried out using the continuous values from 120 heterologous hybridization experiments, where each value corresponds to the (log_26695_-log_heterol.strain_) value for each tested gene (see materials & methods). Colors of the line range from blue, if the gene is present, to red, if absent. The range of intermediate colors reflects the degree of hybridization and thus homology, but also the redundancy of the tested genes. This figure represents the clustering based on the complete set of 254 genes.

Hybridization results for the 120 studied DNAs used as a probe and the home-made macroarrays derived from the reference strain 26695 are presented in Additional file 1 (data based on the binary presence/absence analyses) and Figure 1 (data based on the multidimensional analysis of continuous values, see material and methods). Both presentations illustrate the distribution of each of the 254 genes (213 non-ubiquitous, and 41 ubiquitous, used for normalization) with respect to associated diseases. Each strain hybridization profile (Figure 1) is represented by a series of vertically aligned bar charts, whereas the horizontal lines represent each of the 254 genes. Each strain exhibited a unique profile. The most striking features were related to the distribution of the *cag*PAI genes: almost all *H. pylori *strains associated with metaplasia harbored a complete *cag*PAI, a result consistent with findings by Nilsson et al. [26]. However, a complete *cag*PAI was present in 70% of duodenal ulcer strains, and in 50% of chronic gastritis and of MALT lymphoma strains, confirming previously published findings for isolates collected in the West [27].

**Figure 2:**
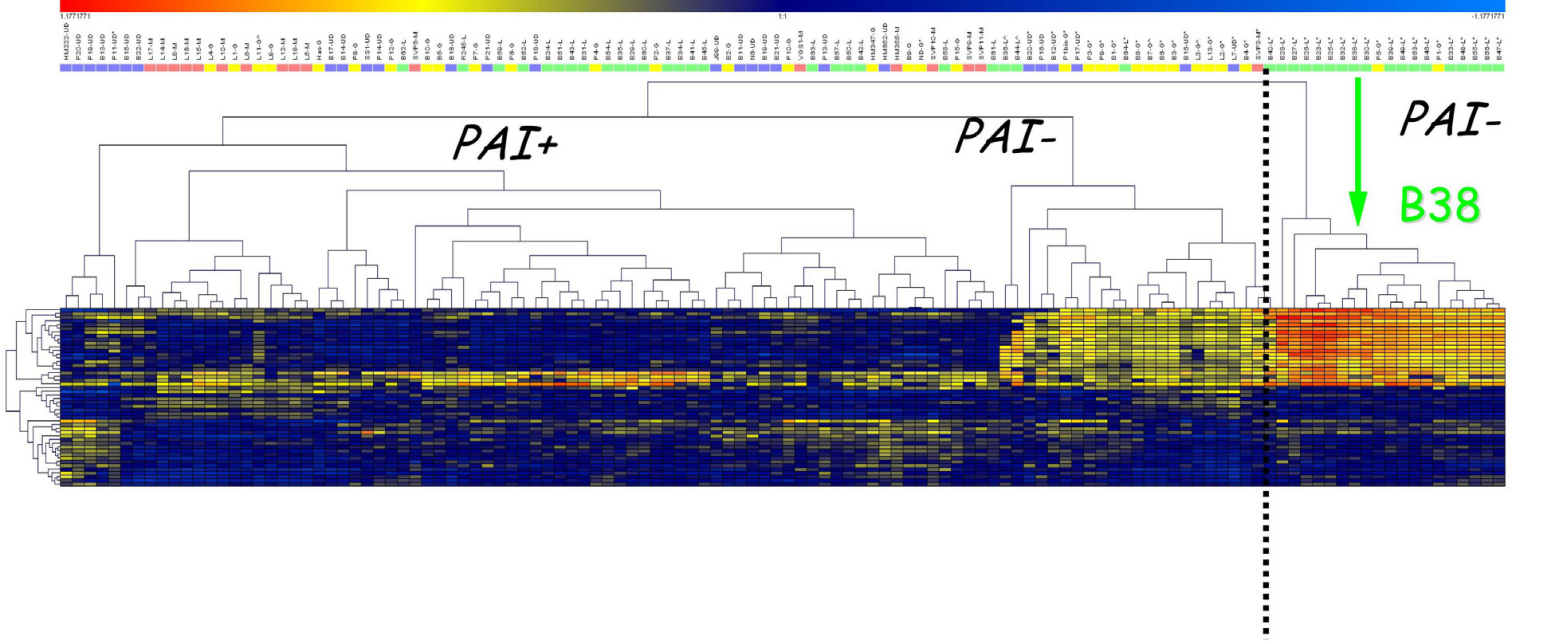
**Hybridization reactions on a DNA macroarray membrane: clustering based on the 48 most discriminatory genes identified as key combinations of variables (genes/axes) from Principal Component Analysis**. These 48 genes are labeled in Addional file 1.

Hierarchical clustering of the continuous values derived from the hybridization experiments of 120 French clinical isolates presenting different disease characteristics was performed (Figure 1). This allowed us to visualize a branch clustering almost exclusively isolates associated with MALT lymphoma. Furthermore, principal component analysis allowed us to identify a combination of 48 genes (Additional file 1), which proved to be the most informative during multidimensional analysis. We then performed hierarchical clustering based on the values of these 48 genes (Figure 2). Two main branches were detected, one consisting of a distinct cluster of 20 isolates, all totally deprived of the *cag*PAI. Eighteen of the isolates were associated with MALT lymphoma and two with gastritis. Interestingly, none of the peptic ulcer or metaplasia isolates clustered in this branch. The second branch splits into two main clusters, one corresponding to isolates that totally or partially lack *cag*PAI genes mostly associated with gastritis and the other clustering isolates associated with other diseases.

To clarify the genetic determinism of the MALT lymphoma strains, we selected one strain that was representative of the MALT lymphoma *cag*PAI minus branch and determined its genome sequence. We selected strain B38, which was isolated from a 62-year-old man suffering from MALT lymphoma. It fulfilled various requirements: i) it belonged to the hpEurope phylogenetic branch according to MLST analysis (Suerbaum, personal communication), a property that was consistent with the five *Helicobacter *genome sequences previously published (26695, J99, HPAG1, P12, and G27); ii) it was genetically transformable; iii) it was plasmid free, and iv) it was capable of colonizing the mouse gastric mucosa. Its *vac*A status was s2m2 [18].

### Main features of the B38 genome

**Figure 3:**
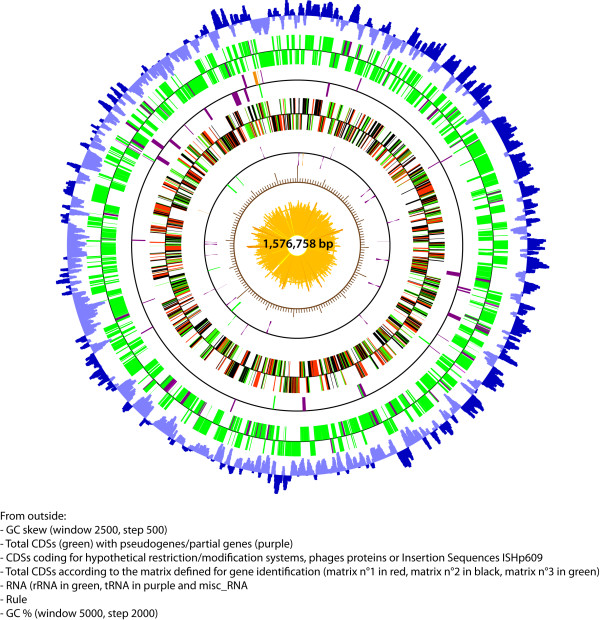
**Genome map of *Helicobacter pylori *strain B38**. From outside to inside: -	GC skew (window 2500, step 500) in blue. -	Total CDSs (green) with pseudogenes/partial genes (purple). -	CDSs coding for hypothetical restriction/modification systems (purple), phage proteins (orange), or insertion sequences (IS*Hp609*) (green). -	Total CDSs according to the matrix defined for gene identification (matrix n°1 in red, matrix N°2 in black, matrix n°3 in green). -	RNA (rRNA in green, tRNA in purple and misc_RNA in red). -	Rule. -	GC% (window 5000, step 2000) in yellow. Red arrow indicates the position of the origin of replication.

The genome of the B38 strain consists of a circular chromosome containing 1,576,758 base pairs (bp) and an average GC content of 39.2% (Figure 3). It is the smallest *H. pylori *genome sequenced to date (Table 1). The B38 genome sequence was first automatically and then manually annotated using the MaGe system [28]http://www.genoscope.cns.fr/agc/mage and was then compared with the other sequenced *H. pylori *genomes. It contains 1,528 CDSs with a coding density (85.0%) similar to that found in the other *Helicobacter *sequenced strains. Among the 1528 CDSs, 1393 were predicted to be protein-coding genes (complete CDSs) with an average length of 971 bp; 135 correspond to partial CDSs, of which 133 are pseudogenes (i.e. 133 fragments representing 62 genes) and two are remnant genes (corresponding to truncated genes for which we cannot find the missing sections in close proximity) (Table 1).

**Table 1:** Summary of comparative features of *Helicobacter *genomes

Features of the strains	B38	26695	J99	**HPAG1**^**a**^	Shi470	**G27**^**a**^	**P12**^**a**^	**H. a Strain**^**a **^**Sheeba**	H. h Strain ATCC 51449
cagPAI	**NEG**	POS	POS	POS	POS	POS	POS	HacGI	HHGI1
									
Size (bp)	**1,576,758**	1,667,867	1,643,831	1,596,366	1,608,547	1,652,982	1,673,813	1,553,927	1,799,146
(G+C) content (%)	**39.2**	38.9	39.2	39.1	38.9	38.9	38.8	38.2	35.9
									
Total CDSs (nb)^b^	**1,528**	1,637	1,543	1,539	1,592	1,611	1,639	1,696	1,851
Complete CDSs (nb)^b^	**1,393**	1,501	1,446	1,441	1,473	1,469	1,505	1,397	1,824
Average length (bp)^b^	**971**	964	988	971	955	954	957	933	914
Coding density (%)^b^	**85.0**	86.3	86.6	87.3	87.2	84.6	85.8	83.6	92.3
Partial CDSs (nb)^b^	**135**	136	97	98	119	142	134	299	27
Truncated genes (nb)^b^	**2**	9	10	7	4	7	7	11	11
Pseudogenes (nb)^b^	**133(8.7%)**^c^	127(7.8%)	87(5.6%)	91(5.9%)	115(7.2%)	135(8.4%)	127(7.8%)	288(17%)	16(0.9%)
Fragmented pseudogenes (nb)^b^	**62(4%)**^**d**^	61(3.7%)	38(2.8%)	43(2.8%)	52(3.2%)	64(3.9%)	56(3.4%)	81(4.8%)	8(0.4%)
									
tRNA (nb)	**36**	36	36	36	36	36	36	36	37
Ribosomal RNA genes									
23S (nb)	**2**	2	2	2	2	2	2	2	1
16S (nb)	**2**	2	2	2	2	2	2	2	1
5S (nb)	**3**	3	2	2	2	3	2	2	1
									
IS-types (ORFs number)	**20IS*Hp609 (*5)**	17 IS*606 * (1) IS*605* (5) IS*200* (1)	6 IS*606 * (1 remnant) IS*Hp609 * (1 remnant)	7 IS*606 * (2 remnant) IS*Hp609* (1)	5 IS*606 * in 3 fragments	9 IS*605* (4)	1	13 IS*Ha1152* (2) IS*Ha1942* (1) IS*Ha1675* (1)	2IS*Hp609 * (1 remnant)

^a^These genomes have got a 9,369 bp (HPAG1), a 10,031 bp (G27), a 10,225 bp (P12), a 3,661 bp (Sheeba) plasmid and a 10,031 bp (G27) and a 10,225 bp (P12). Plasmids were not counted

^b^Revised number with the MaGe system and manual curation

^c^Percentage of fragments of genes/total CDSs

^d^Percentage of fragmented genes/total CDSs

^e ^Number of copies

Of the 1,528 annotated CDSs, a function was assigned to 989 CDSs (64.7%). For 784 of them (79.3%), a function was experimentally demonstrated either in the *Helicobacter *species (188, 12.3%) or in another organism (596, 39%). Two hundred and five CDSs (20.7% of 989) received a function based on the presence of a conserved amino acid motif, a structural feature, or limited homology. A total of 378 CDSs have homologs in previously reported sequences of the genus *Helicobacter *(43.6% of 378), in the epsilon proteobacteria (35.2% of 378), or in other distant bacteria (21.2% of 378). Protein function classification based on the cluster orthologous genes classification (COG) database allowed us to place 1189 of the 1528 CDSs (77.81%) in at least one of the COG functional groups (Table 2): 454 were assigned to cellular processes and signaling systems, 342 to information storage and processing, while 595 were involved in metabolism. The B38 genome exhibits the highest percentage of CDSs associated with a COG group (77.97% *vs *73.38% for 26695, 76.48% for J99, 76.15% for HPAG1 and, 73.49 for Shi470), with the number of CDSs involved in defense mechanisms slightly higher than in the other sequenced *Helicobacter *strains.

**Table 2:** Automatic distribution of protein functions, based on the COG classification, between *Helicobacter *strains

**Species and strains**		***H. p *B38**		***H. p *26695**		***H. p *J99**		***H. p *HPAG1**		***H. p *Shi470**		***H. p *G27**		***H. p *P12**		***H. acinonychis***		***H. hepaticus***	
**CELLULAR PROCESSES AND SIGNALING**																			
																			
Cell cycle control, cell division, chromosome partitioning	D	39	2.55%	39	2.38%	38	2.46%	34	2.21%	38	2.39%	37	2.30%	39	2.38%	37	2.10%	33	1.78%
Cell wall/membrane/envelope biogenesis	M	109	7.13%	106	6.48%	105	6.81%	105	6.82%	104	6,53%	108	6.70%	106	6.47%	109	6.20%	134	7.24%
Cell motility	N	65	4.26%	58	3.54%	60	3.89%	57	3.70%	55	3.46%	59	3.66%	61	3.72%	57	3.24%	68	3.68%
Posttranslational modification, protein turnover, chaperones	O	71	4.65%	74	4.52%	75	4.86%	74	4.81%	77	4.84%	76	4.72%	76	4.64%	73	4.15%	85	4.60%
Signal transduction mechanisms	T	54	3.53%	52	3.18%	56	3.63%	47	3.05%	46	2.89%	51	3.17%	57	3.48%	46	2.62%	68	3.68%
Intracellular trafficking, secretion, vesicular transport	U	59	3.86%	76	4.64%	73	4.73%	66	4.29%	71	4.46%	74	4.59%	83	5.06%	56	3.18%	60	3.24%
Defense mechanisms	**V**	**57**	**3.73%**	**52**	**3.18%**	**54**	**3.50%**	**53**	**3.44%**	**51**	**3.20%**	**53**	**3.29%**	**52**	**3.17%**	**42**	**2.39%**	**38**	**2.05%**
Extracellular structures	W	0	0.00%	0	0.00%	0	0.00%	1	0.07%	1	0.06%	1	0.06%	0	0	0	0%	1	0.05%
Cytoskeleton	Z	0	0.00%	0	0.00%	0	0.00%	0	0.00%	0	0.00%	0	0	0	0	0	0%	1	0.05%
**INFORMATION STORAGE AND PROCESSING**																			
Chromatin structure and dynamics	B	1	0.07%	1	0.06%	0	0	1	0.07%	1	0.06%	1	0.06%	1	0.06%	0	0%	1	0.05%
Translation, ribosomal structure, biogenesis	J	134	8.77%	138	8.43%	141	9.14%	137	8.90%	138	8.67%	136	8.44%	139	8.48%	134	7.62%	148	8.00%
Transcription	K	53	3.47%	44	2.69%	46	2.98%	48	3.12%	43	2.70%	49	3.04%	49	2.99%	43	2.45%	59	3.19%
Replication, recombination and repair	**L**	**154**	**10.08%**	**177**	**10.69%**	**155**	**10.05%**	**149**	**9.68%**	**150**	**9.42%**	**171**	10.62%	**163**	**9.95%**	**155**	**8.82%**	**100**	**5.41%**
**METABOLISM**																			
Energy prodcution and conversion	C	90	5.89%	89	5.44%	87	5.64%	93	6.04%	93	5.84%	92	**5.71%**	93	5.67%	87	4.95%	118	6.38%
Amino acid transport	E	151	9.88%	145	8.86%	152	9.85%	146	9.49%	153	9.61%	150	9.31%	150	9.15%	160	9.10%	200	10.81%
Nucleotide transport	F	46	3.01%	45	2.75%	47	3.05%	45	2.92%	44	2.76%	47	2.92%	47	2.87%	47	2.67%	58	3.14%
Carbohydrate transport	G	60	3.93%	56	3.42%	57	3.69%	55	3.57%	63	3.96%	55	3.41%	59	3.60%	59	3.35%	76	4.11%
Coenzyme transport	H	75	4.91%	73	4.46%	75	4.86%	75	4.87%	75	4.71%	75	4.66%	76	4.64%	67	3.81%	87	4.70%
Lipid transport	I	50	3.27%	49	2.99%	49	3.18%	50	3.25%	47	2.95%	51	3.17%	51	3.11%	47	2.67%	54	2.92%
Inorganic ion transport	P	97	6.35%	94	5.74%	100	6.48%	94	6.11%	103	6.47%	97	6.02%	98	5.98%	95	5.40%	125	6.76%
Secondary matabolites biosynthesis, transport	Q	26	1.70%	25	1.53%	25	1.62%	23	1.50%	23	1.45%	22	1.37%	26	1.59%	24	1.36%	37	2.00%
**POORLY CHARACTERIZED**																			
General function prediction only	R	174	11.39%	173	10.57%	178	11.54%	160	10.40%	174	10.93%	168	10.43%	175	10.68%	166	9.44%	234	12.65%
Function unknown	S	84	5.50%	80	4.89%	71	4.60%	78	5.07%	70	4.40%	84	5.21%	81	4.94%	70	3.98%	113	6.11%
**CDS Not Classified to any COG (Number/%)**		**339/22.2**		**435/26.6**		**363/23.5**		**367/23.9**		**422/26.5**		**395/24.5**		**440/26.9**		**536/31.6**		**479/25.9**	
TOTAL CDS*		**1528**		**1637**		**1543**		**1539**		**1592**		**1611**		**1639**		**1696**		**1850**	
**%CDS at least in one COG**		**77.81%**		**73.43%**		**76.47%**		**76.15%**		**73.49%**		**75.48%**		**73.15%**		**68.40%**		**74.11%**	

*The CDSs were manually curated in the MaGe system for the elimination of artifacts.

There are a significant number of restriction/modification systems present in *H. pylori*; their composition and activity have been shown to be strain-specific [29]. In the B38 strain, 63 CDSs were involved in restriction/modification systems. Among them, 30 elements were fragmented into pseudogenes corresponding to 12 potential genes, and three elements appeared to be partial genes (Additional file 2). Thus, the proportion of potentially active genes (52%) appeared to be higher in B38 than in strains J99 and 26695, in which only 30% of type II R-M systems were reported to be functional [30].

The B38 genome harbors five complete copies of the four-gene insertion sequence IS*Hp609*. This insertion sequence was frequently found in *H. pylori *strains from Europe, Americas, India and Africa, but was almost always absent in strains from East Asia [31]. Three of the four genes (*orf1*, *orf2*, *ORFA*) demonstrated 100% of identity in the five B38 IS*Hp609 *copies, whereas *ORFB *from one of the five B38 IS*Hp609 *copies (HELPY1334) exhibited a single mutation. Among the sequenced genomes (Table 1), a single and complete copy of this element was found in strain HPAG1, but it differed slightly from that found in B38 (6, 8, and 9 mutations are present in orf1, ORFA, and ORFB of HPAG1, respectively). This consistency in the five copies of IS*Hp609 *in B38 indicated that it has been acquired very recently, and that it is probably an active element that is capable of transposition, a property never experimentally demonstrated for a transposable element in *H. pylori*.

Another property associated with the B38 genome relates to the complete absence of four of the 45 genes encoding outer membrane proteins (OMPs) from the four conserved OMP families (Hop, Hor, Hof et Hom) (Additional file 3). B38 lacks *bab*B, *bab*C, *sab*B, and *hom*B, four OMPs known to play a major role in adhesion to gastric epithelial cells and possibly in long-term persistence of strains in the human gastric mucosa when associated with peptic ulcer diseases or gastric metaplasia [32]. B38 lacks a high number of adhesin genes among the sequenced genomes.

### Comparative genomics and genome evolution

**Figure 4:**
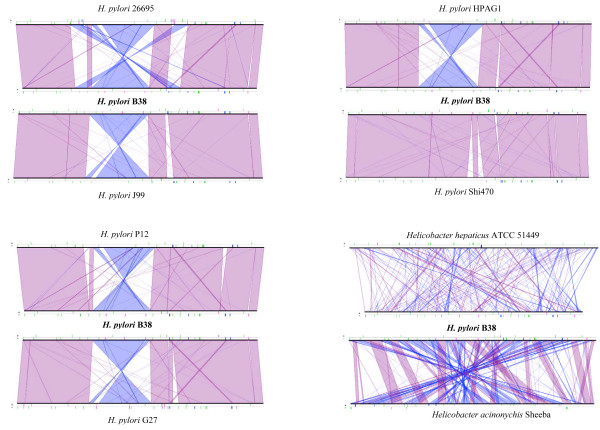
**Synteny lineplot pair-wise analyses between B38 and the *H. pylori *strain 26695, J99, HPAG1, Shi470, P12, G27, *Helicobacter hepaticus*, or *Helicobacter acinonychis***.

We then analyzed the genomic rearrangements through pair-wise genomic synteny comparisons between B38 and the eight published *Helicobacteriaceae *genomes. For five of the isolates (namely, 26695, J99, G27, P12, HPAG1), we confirmed the previously reported relative colinearity of the *H.pylori *genomes. This colinearity is mainly interrupted by insertion elements, the *cag*PAI, and genes encoding hypothetical proteins [33]. However, unexpectedly, conserved synteny highlighted an almost complete colinearity never described so far, between B38 and Shi470 (Figure 4). Shi470 is a clinical isolate from the gastric antrum of an Amerindian resident of a remote Amazonian village in Peru, and was thought to be related to strains from East Asia [RefSeq:NC_010698]. This unexpected absence of major genomic rearrangements between the two genomes prompted us to compare the genome of these two isolates more closely, as a way of better understanding *H. pylori *genome evolution. B38 lacks 174 Shi470 genes, of which 70 genes cluster in three insertion blocks: one corresponds to the well characterized *cag*PAI; another to a block of 33 CDSs, mainly remnants from a conjugative plasmid (presence of TraG, VirB11, toposiomerase I, ComB3, homologs of conjugal plasmid transfer system); and the third corresponds to a block that includes 7 CDSs encoding hypothetical proteins, as well as one CDS encoding an exodeoxyribonuclease subunit which is unique to the Shi 470 isolate.

Conversely, loss of synteny was also due to the presence of 110 CDSs in B38 that were not present in Shi470. Forty-three of these CDSs appeared as clusters within eight loci. Twenty corresponded to IS*Hp609 *(5 complete and conserved copies of IS*Hp609 *each comprising *orf1*, *orf2*, *ORFA *and *ORFB*) [31], which interrupts HELPY0571, HELPY0700 (both encoding restriction/modification systems), HELPY0838 (encoding a putative Rad50 ATPase), HELPY1330 (encoding a putative glycosyl-transferase), and HELPY1529 (a HAC prophage II protein homolog). In addition to these five IS*Hp609 *insertions, loss of synteny was also due to the presence of CDSs in four other loci: i) a cluster of seven genes (HELPY1520 to HELPY1525 and HEPLY1527, HELPY1528 to HELPY1533) encoding HacII prophage-like proteins similar to those found in *H. acinonychis *strain Sheeba [34]; however, the size of the prophage is much larger (32 CDS) in this species, suggesting that the prophage in B38 has been deleted, possibly following the insertion of one copy of IS*Hp609*; ii) a cluster of six genes encoding hypothetical proteins of unknown function (HELPY0051 to HELPY0056); iii) a cluster of three CDSs that are absent in Shi470, HPAG1, J99, P12, and G127, but present in strain 26695, of which two encode alginate-O-acetylation proteins (HELPY0497-498); iv) a cluster of seven CDSs that encode a putative helicase (HELPY0989) and a putative serine kinase (HELPY0990), two functional proteins not found in all of the other sequenced strains.

### *H. pylori *core genome and strain-specific genes

BLAST score ratio analyses and comparisons between the B38 strain and the six other sequenced genomes, which were analyzed and revised through the MaGe system (Table 1), allowed us to establish that the core of the *H. pylori *genome consists of 1,275 CDSs. This number is slightly higher than that recently published by McClain and colleagues who identified 1,237 genes, as it takes into account additional CDSs detected by the MaGe system [35]. This number is lower than that calculated from data presented in Additional file 1 (1,358 genes) based on the macroarray hybridization analysis of 120 isolates. This approach overestimated the number of ubiquitous CDSs, as all small CDS (<350 bp) from the 26695 strain genome were excluded from the analysis, and thus were systematically counted as ubiquitous CDSs.

To identify strain-specific genes present in the B38 strain but absent from the other sequenced strains, we studied the putative orthologous relationship between two genomes *i.e. *gene couples who satisfy Bi-directional Best Hit (BBH) criteria. Criteria included a minimum of 30% sequence identity and 80% of the length of the smallest protein (Additional file 4). Only 16 CDSs were found to be unique to the B38 strain: nine seemed to be complete and thus putatively functional; six were shown to encode the putative HacII prophage-like proteins (HELPY1521-1522-1523-1524-1525-1527); three were found to encode hypothetical proteins (HELPY0409, HELPY0645 and HELPY0996), and seven corresponded to fragments of genes (partial genes) coding for either conserved hypothetical proteins, prophage-like sequences or for a restriction enzyme. Using the same methodology, we looked for genes that were present in the various *H. pylori *strains and absent in B38 (Additional file 5). If compared pair-wise, the number of CDSs absent in B38 was between 105 and 175. The only genes that were found to be exclusively absent in B38 corresponded to those of the *cag*PAI (Additional file 5), the well-known cluster of genes involved in the induction of a strong inflammatory response.

### Specific properties associated with the genomes of strains belonging to the MALT lymphoma PAI minus cluster

Of the 19 strains belonging to the MALT lymphoma PAI minus cluster, all 19 contained the *vac*Am2 allele; 16 exhibited an s2m2 genotype, indicating that they encode a non-functional cytotoxin, and three exhibited an s1m2 genotype [18]. We then investigated whether the properties found to be unique to strain B38 are shared by the strains belonging to the cluster of the MALT lymphoma PAI-minus cluster. The search for the presence of the HacII-like prophage was done through hybridization using internal fragments of HELPY1521, HELPY1525, and HELPY1526 as probes. Four of the 19 strains (21%, including B38) of the MALT lymphoma PAI minus cluster, contained HacII prophage-like sequences. By contrast, 1/24 (4%) strains isolated from patients with MALT lymphoma containing *cag*PAI, 2/33 (6%) strains from patients suffering from gastritis and 2/27 strains (7.4%) from those with duodenal ulcers contained HacII prophage-like sequences. Furthermore, the presence of the two adjacent HELPY0989 and HELPY0990 genes encoding a helicase and a serine kinase, respectively, not previously found in the other sequenced genomes as functional proteins were found in three of the 19 strains (16%) of the B38 cluster. These two genes were not detected in the other MALT lymphoma strains (*cag*PAI positive), nor within the 22 isolates associated with gastritis and peptic ulcers. Finally, three clustered conservative mutations in *glmM *(HELPY*0072 *- Ala_332_, Leu_333_), leading to the absence of amplification of the 294-bp internal fragment of the phosphoglucosamine mutase-encoding gene [36], were observed in five of the 19 MALT lymphoma PAI minus isolates (26%). However, these mutations were not found in any of the 120 clinical isolates of this study, nor were they found in more than 400 *H. pylori *isolates associated with gastritis, peptic ulcers or metaplasia that were tested with identical oligonucleotides (personal data). These conservative mutations may be indicative of a selective pressure to maintain these mutations, together with a property encoded by a gene present in close proximity to *glmM*, a property that has yet to be identified. Thus, although none of the unique properties of B38 were shared by all MALT strains of the cluster, characterizing a *cagPAI *minus isolate containing either *glmM *mutations or HELPY0989-0990 genes may be predictive of MALT lymphoma, as these two characteristics were found exclusively among the strains of this cluster.

## Conclusion

The study was initiated with the aim of gaining insight into the existence of bacterial determinism for gastric extra-nodal marginal zone B-cell MALT lymphoma. DNA hydridization against the whole genome of 120 clinical isolates revealed a cluster of 19 *H. pylori *strains, all completely deprived of *cag*PAI sequences originating from patients with MALT lymphoma. We sequenced the genome of strain B38, a representative of this cluster, and describe the first genome sequence of a *cag*PAI minus *H. pylori *strain. The absence of the *cag*PAI, including that of several non-ubiquitous genes, makes the B38 genome the smallest *H. pylori *genome described to date. The *cagPAI *minus B38 strain lacks a functional cytotoxin (*vac*As2m2) as well as genes encoding the major adhesion factors (absence of *bab*B, *bab*C, *sab*B, and *hom*B); thus, compared with well-known pro-inflammatory *H. pylori *isolates, it appears to be deprived of all known pathogenic determinants, but is nonetheless associated with gastric neoplasia. Further investigation is required to fully understand the difference in fitness between these strains with low pro-inflammatory profiles and the human host factors that may play a significant role in the development of gastric MALT lymphoma.

## Methods

### *H. pylori *strains, and growth

We examined 120 *H. pylori *strains isolated from patients from different areas of France enrolled in 3 multi-center studies carried out by 1) the *Groupe d'Etude Français des Helicobacter *(G.E.F.H.), 2) *the Groupe d'Etude Français des Lymphomes Digestifs *(G.E.L.D.) [37] and of the *Fédération Française de Cancérologie Digestive *(F.F.C.D.) [38], and 3) the *Groupe d'Etude des Lymphomes de l'Adulte *(G.E.L.A.). Criteria for patient inclusion were age (>55 years), suffering from chronic gastritis (n = 33), duodenal ulcer without intestinal metaplasia (27), intestinal metaplasia without ulcer (n = 17). We identified 43 strains from patients with gastric MALT lymphoma. *H. pylori *was isolated from one biopsy specimen following biopsy homogenization and culture under microaerophilic conditions (5-6% 0_2_, 8-10% CO_2_, 80-85% N_2_) on blood agar medium (BA; Oxoid blood agar base N°2) supplemented with 10% horse blood, as reported previously [39]. One colony was selected at random from each primary culture; it was then sub-cultured and used to prepare chromosomal DNA. This DNA was extracted from 48-hour-old confluent cells using the QIAamp Tissue kit (Qiagen, Chatsworth, CA) according to the manufacturer's recommendations.

### In house DNA macroarray membrane preparation

A total of 254 PCR products were amplified in four 96-well microtiter plates, corresponding to 41 ubiquitous and 213 non-ubiquitous genes from the genome of strain 26695 as previously described [39]. Briefly, amplification reactions were performed in 2 × 100 μl reaction volumes, in which 2 μl of DNA corresponding to the recombinant plasmid containing the full-length CDS (CoDing Sequence) inserted into the pILL570-derivative vector was used as template. Each PCR product was sequenced to confirm the identity of the gene, and was then spotted in triplicate onto a nylon membrane (Qfilter, Genetix 22.2 × 22.2 cm, N+) using a Qpix robot (Genetix). Denaturated 26695 genomic DNA was spotted in triplicate at the four corners of the membrane (positive controls) and seven squares were left empty as negative controls. Following spot deposition, membranes were fixed for 15 minutes in 0.5 M NaOH 1.5 M NaCl, washed briefly in distilled water, and stored wet at -20°C until use [39].

Aliquots of 250 μl of DNA were labeled by random priming with 2 μl of ^33^P-dCTP. Labeling was performed for 3 hours at room temperature. Unincorporated radionucleotides were removed by purification on Quick Spin Sephadex G-25 columns (Roche Diagnostics). Immediately before being used for hybridization experiments, the sonicated, labeled, and purified chromosomal DNA was heat-denaturated and cooled on ice. Hybridization was conducted in 5 ml prewarmed (65°C) hybridization mixtures containing the heat denaturated probe, with overnight incubation. Membranes were then washed and exposed for 25 hours to a phosphoimager screen (Molecular Dynamics).

Screens were scanned on a Storm 860 machine (Molecular Dynamics). Image analysis and quantification of hybridization intensities for each spot were performed using the Xdots Reader program (COSE) and determined in pixels [39]. The intensity of the background surrounding each spot was substracted from that of each of the spots. Twenty-one homologous hybridizations were performed. The average intensity of the 41 ubiquitous genes was calculated for each reference array. This number served to allocate a reference array to each heterologous hybridization (average of the ubiquitous spots from the heterologous and the homologous reference hybridizations were not significantly different, Student's test), to calculate the ratio used for normalization. Following normalization, the data were analyzed by attributing a binary score (presence/absence - Additional file 1) or by multidimensional analysis based on continuous intensity values (Figure 1 and Figure 2). To define the cutoff ratio for the presence/absence of a gene, we analyzed the results for the sequenced *H. pylori *J99 DNA hybridized with *H. pylori *26695; the threshold for the presence of a gene was defined as >0.25. The multidimensional analyses (Genesis software) for the hierarchical clustering as well as for the Principal Component Analysis were performed using the 254 continuous values from the 120 heterologous hybridization experiments, each corresponding to (log_10_normalized intensity values of strain 26695) minus (log_10_normalized intensity values of the heterologous strain) (*i.e. *log_26695_-log_heterol.strain_).

### Sequencing and annotation of the B38 genome

Genomic DNA was randomly sheared by nebulization (HydroShear, GeneMachines) and the ends were enzymatically repaired. *Sma*I fragments (1.5-4 kb) were inserted into plasmid vector pBAM3/*Sma*I (derived from pBluescript KS and constructed by R. Heilig). Large (35-45 kb) DNA fragments generated from partial *BamH*I-restriction were inserted into the cosmid vector pHC79/*BamH*I.

Plasmid DNA was prepared with the TempliPhi DNA sequencing template amplification kit (GE Healthcare-Bio-Sciences). Cosmid DNA was purified with the Montage BAC Miniprep96 Kit (Millipore). Sequencing reactions were performed from both ends of DNA templates using ABI PRISM BigDye Terminator cycle sequencing ready reactions kits and were run on a 3700 or a 3730 xl Genetic Analyzer (Applied Biosystems).

Sequence data base calling was carried out using Phred [40]. Sequences not meeting our production quality criteria (at least 100 bases called with a quality over 20) were discarded. Sequences were screened against plasmid vector and *E. coli *sequences. The traces were assembled using Phrap and Consed [41]. Whole genome shotgun sequencing was performed to ensure approximately 11-fold coverage. Autofinish [42] was used to design primers for improving regions of low quality sequence and for primer walking along templates spanning the gaps between contigs. Several strategies were used to orientate contigs and to enable directed PCR-based approaches to span the gaps between contigs. These strategies included linking isolates and a Blast-based approach, which identified contigs with hits to the *H. pylori *strain 26695 genome. Various combined PCR techniques were used to amplify genomic or cosmid DNA, to close the gaps between the final contigs. Outward-directed primers were designed for each of the contig ends; the primer sequences were subsequently checked and confirmed to be unique to the genome. This combined PCR process required approximately 200 PCR reactions pairing each of the primers. In addition, two cosmid isolates containing a rDNA operon copy each, were completely sequenced by sub-cloning into a pSMART-LC vector (Lucigen Corp.). The error rate was less than 1 error per 10,000 bp in the final assembly. The complete genome sequence was obtained from 40 153 sequences, resulting in 14-fold coverage.

AMIGene software was used to predict which CDSs were likely to encode proteins [43]. The set of predicted genes underwent automatic functional annotation using the set of tools listed in Vallenet *et al*. [28]. All these data (syntactic and functional annotations, results of comparative analysis) are stored in a relational database, called PyloriScope. Manual validation of the automatic annotation was performed using the MaGe (Magnifying Genomes, http://www.genoscope.cns.fr) web-based interface, which allows graphic visualization of the annotations enhanced by the synchronized representation of synteny groups in other genomes chosen for comparison.

### Accession Numbers

The EMBL Nucleotide Sequence Database http://www.ebi.ac.uk/embl accession number for the *H. pylori *strain B38 chromosome is [EMBL:FM991728].

All data and comparative genomics concerning the *H. pylori *B38 genome are stored in PyloriScope http://www.genoscope.cns.fr/agc/mage, a related database that is available to the public.

## Authors' contributions

JMT carried out the macroarrays, the molecular genetic studies, and participated to the genome assembly. CB-E carried out the major part of the manual annotation of the genome together with PL, HDR, and IB. CB and LM carried out to the genome sequencing and assembly. CM, ZR and AL were involved in the automatic annotation, comparative genomics, and administration of the MaGe system. JYC, MAD and SC participated to the home made DNA arrays preparation, and the statistical analyses. CB, AR-F, AC-M, DL, FM and JCD collected the clinical isolates. AL designed the study, analysed the results, and drafted the manuscript. JR analysed the results, and drafted the manuscript. All authors read and approved the final manuscript.

## Supplementary Material

Additional file 1 **List of the 254 genes of *Helicobacter pylori *strain 26695 used for gene amplification and preparation of the home-made macroarray membranes**. Distribution of each gene in the 120 French isolates of this study associated with gastritis (G), duodenal ulcer (DU), gastric MALT lymphoma (MALT) or metaplasia (META). The percentages were based on the binary analysis (presence/absence/) according to the normalization process and the cutoff ratio described in Material ad Methods. "HPXXXX+", genes were designated as ubiquitous genes based on previous comparative analysis [25]; "HPXXXX" are the non-ubiquitous genes; the 48 most discriminatory genes identified as key combinations of variables (genes/axes) from the Principal Component Analysis, which were used for the clustering analysis, are in bold (Figure 2). Click here for file

Additional file 2 **CDSs of B38 strain involved in restriction/modification systems classified according to the gene status**. Click here for file

Additional file 3 **Distribution of the outer membrane proteins (OMPs) encoding genes in the 7 *Helicobacter pylori *genome sequences**. (B38, J99, 26695, HPAG1, Shi470, G27, P12). The genes are classified according to the *hop, hor, hof*, and *hom *gene families. The numbers refer to the name of the CDS in each genome (for example: 0009 in 26695 refers to HP0009, 0007 in B38 refers to HELPY0007). "x" indicates a complete absence of the gene. Two or three names separated by a "/" reveals the presence of a pseudogene. Click here for file

Additional file 4 **Number of CDSs in the B38 strain that are absent in the J99, 26695, HPAG1 or Shi470 *Helicobacter pylori *strains classified by protein functions**. Click here for file

Additional file 5 **Number of CDSs (listed by protein functions) of the *Helicobacter pylori *J99, 26695, HPAG1 and Shi470, G27 and P12 strains that are absent in strain B38 respectively**. * All strains: J99, 26695, HPAG1, Shi470, G27, and P12. ** The number depends on the strain chosen for reference. Click here for file

## References

[B1] Blaser MJ Atherton JC *Helicobacter pylori *persistence: biology and disease J Clin Invest 2004 113 321 333 14755326 10.1172/JCI20925 PMC324548

[B2] Forman D Newell DG Fullerton F Yarnell JW Stacey AR Wald N Sitas F Association between infection with *Helicobacter pylori *and risk of gastric cancer: evidence from a prospective investigation BMJ 1991 302 1302 1305 10.1136/bmj.302.6788.1302 2059685 PMC1670011

[B3] Isaacson P Wright DH Jones DB Malignant lymphoma of true histiocytic (monocyte/macrophage) origin Cancer 1983 51 80 91 10.1002/1097-0142(19830101)51:1<80::AID-CNCR2820510118>3.0.CO;2-0 6336974

[B4] Uemura N Okamoto S Yamamoto S Matsumura N Yamaguchi S Yamakido M Taniyama K Sasaki N Schlemper RJ *Helicobacter pylori *infection and the development of gastric cancer N Engl J Med 2001 345 784 789 10.1056/NEJMoa001999 11556297

[B5] Gerhard M Rad R Prinz C Naumann M Pathogenesis of *Helicobacter pylori *infection Helicobacter 2002 7 Suppl 1 17 23 10.1046/j.1523-5378.7.s1.3.x 12197905

[B6] Parsonnet J Friedman GD Orentreich N Vogelman H Risk for gastric cancer in people with CagA positive or CagA negative *Helicobacter pylori *infection Gut 1997 40 297 301 9135515 10.1136/gut.40.3.297 PMC1027076

[B7] Leunk RD Johnson PT David BC Kraft WG Morgan DR Cytotoxic activity in broth-culture filtrates of *Campylobacter pylori* J Med Microbiol 1988 26 93 99 10.1099/00222615-26-2-93 3385767

[B8] Cover TL Blanke SR *Helicobacter pylori *VacA, a paradigm for toxin multifunctionality Nat Rev Microbiol 2005 3 320 332 10.1038/nrmicro1095 15759043

[B9] Ilver D Arnqvist A Ogren J Frick IM Kersulyte D Incecik ET Berg DE Covacci A Engstrand L Boren T *Helicobacter pylori *adhesin binding fucosylated histo-blood group antigens revealed by retagging Science 1998 279 373 377 10.1126/science.279.5349.373 9430586

[B10] Mahdavi J Sondén B Hurtig M Olfat FO Forsberg L Roche N Angstrom J Larsson T Teneberg S Karlsson KA Altraja S Wadström T Kersulyte D Berg DE Dubois A Petersson C Magnusson KE Norberg T Lindh F Lundskog BB Arnqvist A Hammarström L Borén T *Helicobacter pylori *SabA adhesin in persistent infection and chronic inflammation Science 2002 297 573 578 10.1126/science.1069076 12142529 PMC2570540

[B11] Bai Y Zhang YL Wang JD Lin HJ Zhang ZS Zhou DY Conservative region of the genes encoding four adhesins of *Helicobacter pylori: *cloning, sequence analysis and biological information analysis Di Yi Jun Yi Da Xue Xue Bao 2002 22 869 871 12377603

[B12] Oleastro M Cordeiro R Yamaoka Y Queiroz D Megraud F Monteiro L Menard A Disease association with two *Helicobacter pylori *duplicate outer membrane protein genes, homB and homA Gut Pathog 2009 1 12 10.1186/1757-4749-1-12 19545429 PMC2706848

[B13] Walz A Odenbreit S Mahdavi J Boren T Ruhl S Identification and characterization of binding properties of *Helicobacter pylori *by glycoconjugate arrays Glycobiology 2005 15 700 708 10.1093/glycob/cwi049 15716466

[B14] Backstrom A Lundberg C Kersulyte D Berg DE Boren T Arnqvist A Metastability of *Helicobacter pylori *bab adhesin genes and dynamics in Lewis b antigen binding Proc Natl Acad Sci USA 2004 101 16923 16928 10.1073/pnas.0404817101 15557006 PMC534723

[B15] Franco AT Israel DA Washington MK Krishna U Fox JG Rogers AB Neish AS Collier-Hyams L Perez-Perez GI Hatakeyama M Whitehead R Gaus IC O'Brien DP Romero-Gallo J Peek RM Jr Activation of beta-catenin by carcinogenic *Helicobacter pylori* Proc Natl Acad Sci USA 2005 102 10646 10651 10.1073/pnas.0504927102 16027366 PMC1180811

[B16] Broutet N Moran A Hynes S Sakarovitch C Megraud F Lewis antigen expression and other pathogenic factors in the presence of atrophic chronic gastritis in a European population J Infect Dis 2002 185 503 512 10.1086/339016 11865403

[B17] Koehler CI Mues MB Dienes HP Kriegsmann J Schirmacher P Odenthal M *Helicobacter pylori *genotyping in gastric adenocarcinoma and MALT lymphoma by multiplex PCR analyses of paraffin wax embedded tissues Mol Pathol 2003 56 36 42 10.1136/mp.56.1.36 12560462 PMC1187288

[B18] Lehours P Menard A Dupouy S Bergey B Richy F Zerbib F Ruskone-Fourmestraux A Delchier JC Megraud F Evaluation of the association of nine *Helicobacter pylori *virulence factors with strains involved in low-grade gastric mucosa-associated lymphoid tissue lymphoma Infect Immun 2004 72 880 888 10.1128/IAI.72.2.880-888.2004 14742532 PMC321584

[B19] Lehours P Zheng Z Skoglund A Megraud F Engstrand L Is there a link between the lipopolysaccharide of *Helicobacter pylori *gastric MALT lymphoma associated strains and lymphoma pathogenesis? PLoS One 2009 4 e7297 10.1371/journal.pone.0007297 19806222 PMC2752801

[B20] Tomb JF White O Kerlavage AR Clayton RA Sutton GG Fleischmann RD Ketchum KA Klenk HP Gill S Dougherty BA Nelson K Quackenbush J Zhou L Kirkness EF Peterson S Loftus B Richardson D Dodson R Khalak HG Glodek A McKenney K Fitzegerald LM Lee N Adams MD Hickey EK Berg DE Gocayne JD Utterback TR Peterson JD Kelley JM The complete genome sequence of the gastric pathogen *Helicobacter pylori* Nature 1997 388 539 547 10.1038/41483 9252185

[B21] Oh JD Kling-Backhed H Giannakis M Xu J Fulton RS Fulton LA Cordum HS Wang C Elliott G Edwards J Mardis ER Engstrand LG Gordon JI The complete genome sequence of a chronic atrophic gastritis *Helicobacter pylori *strain: evolution during disease progression Proc Natl Acad Sci USA 2006 103 9999 10004 10.1073/pnas.0603784103 16788065 PMC1480403

[B22] Baltrus DA Amieva MR Covacci A Lowe TM Merrell DS Ottemann KM Stein M Salama NR Guillemin K The complete genome sequence of *Helicobacter pylori *strain G27 J Bacteriol 2009 191 447 448 10.1128/JB.01416-08 18952803 PMC2612421

[B23] Salama N Guillemin K McDaniel TK Sherlock G Tompkins L Falkow S A whole-genome microarray reveals genetic diversity among *Helicobacter pylori *strains Proc Natl Acad Sci USA 2000 97 14668 14673 10.1073/pnas.97.26.14668 11121067 PMC18976

[B24] Gressmann H Linz B Ghai R Pleissner KP Schlapbach R Yamaoka Y Kraft C Suerbaum S Meyer TF Achtman M Gain and loss of multiple genes during the evolution of *Helicobacter pylori* PLoS Genet 2005 1 e43 10.1371/journal.pgen.0010043 16217547 PMC1245399

[B25] Raymond J Thiberge JM Kalach N Bergeret M Dupont C Labigne A Dauga C Using macro-arrays to study routes of infection of *Helicobacter pylori *in three families PLoS One 2008 3 e2259 10.1371/journal.pone.0002259 18493595 PMC2375058

[B26] Nilsson C Sillen A Eriksson L Strand ML Enroth H Normark S Falk P Engstrand L Correlation between cag pathogenicity island composition and *Helicobacter pylori*-associated gastroduodenal disease Infect Immun 2003 71 6573 6581 10.1128/IAI.71.11.6573-6581.2003 14573679 PMC219608

[B27] Ali M Khan AA Tiwari SK Ahmed N Rao LV Habibullah CM Association between cag-pathogenicity island in *Helicobacter pylori *isolates from peptic ulcer, gastric carcinoma, and non-ulcer dyspepsia subjects with histological changes World J Gastroenterol 2005 11 6815 6822 16425389 10.3748/wjg.v11.i43.6815 PMC4725035

[B28] Vallenet D Labarre L Rouy Z Barbe V Bocs S Cruveiller S Lajus A Pascal G Scarpelli C Medigue C MaGe: a microbial genome annotation system supported by synteny results Nucleic Acids Res 2006 34 53 65 10.1093/nar/gkj406 16407324 PMC1326237

[B29] Xu Q Morgan RD Roberts RJ Blaser MJ Identification of type II restriction and modification systems in *Helicobacter pylori *reveals their substantial diversity among strains Proc Natl Acad Sci USA 2000 97 9671 9676 10.1073/pnas.97.17.9671 10944229 PMC16923

[B30] Lin LF Posfai J Roberts RJ Kong H Comparative genomics of the restriction-modification systems in *Helicobacter pylori* Proc Natl Acad Sci USA 2001 98 2740 2745 10.1073/pnas.051612298 11226310 PMC30209

[B31] Kersulyte D Kalia A Zhang M Lee HK Subramaniam D Kiuduliene L Chalkauskas H Berg DE Sequence organization and insertion specificity of the novel chimeric IS*Hp609 *transposable element of *Helicobacter pylori* J Bacteriol 2004 186 7521 7528 10.1128/JB.186.22.7521-7528.2004 15516563 PMC524915

[B32] Colbeck JC Hansen LM Fong JM Solnick JV Genotypic profile of the outer membrane proteins BabA and BabB in clinical isolates of *Helicobacter pylori* Infect Immun 2006 74 4375 4378 10.1128/IAI.00485-06 16790815 PMC1489689

[B33] Alm RA Trust TJ Analysis of the genetic diversity of *Helicobacter pylori: *the tale of two genomes J Mol Med 1999 77 834 846 10.1007/s001099900067 10682319

[B34] Eppinger M Baar C Linz B Raddatz G Lanz C Keller H Morelli G Gressmann H Achtman M Schuster SC Who ate whom? Adaptive Helicobacter genomic changes that accompanied a host jump from early humans to large felines PLoS Genet 2006 2 e120 10.1371/journal.pgen.0020120 16789826 PMC1523251

[B35] McClain MS Shaffer CL Israel DA Peek RM Jr Cover TL Genome sequence analysis of *Helicobacter pylori *strains associated with gastric ulceration and gastric cancer BMC Genomics 2009 10 3 10.1186/1471-2164-10-3 19123947 PMC2627912

[B36] Kansau I Raymond J Bingen E Courcoux P Kalach N Bergeret M Braimi N Dupont C Labigne A Genotyping of *Helicobacter pylori *isolates by sequencing of PCR products and comparison with the RAPD technique Res Microbiol 1996 147 661 669 10.1016/0923-2508(96)84023-X 9157493

[B37] Levy M Copie-Bergman C Traulle C Lavergne-Slove A Brousse N Flejou JF de Mascarel A Hemery F Gaulard P Delchier JC Conservative treatment of primary gastric low-grade B-cell lymphoma of mucosa-associated lymphoid tissue: predictive factors of response and outcome Am J Gastroenterol 2002 97 292 297 10.1111/j.1572-0241.2002.05460.x 11866264

[B38] Lehours P Dupouy S Bergey B Ruskone-Foumestraux A Delchier JC Rad R Richy F Tankovic J Zerbib F Megraud F Menard A Identification of a genetic marker of *Helicobacter pylori *strains involved in gastric extranodal marginal zone B cell lymphoma of the MALT-type Gut 2004 53 931 937 10.1136/gut.2003.028811 15194637 PMC1774103

[B39] Raymond J Thiberge JM Chevalier C Kalach N Bergeret M Labigne A Dauga C Genetic and transmission analysis of *Helicobacter pylori *strains within a family Emerg Infect Dis 2004 10 1816 1821 15504269 10.3201/eid1010.040042 PMC3323258

[B40] Ewing B Green P Base-calling of automated sequencer traces using phred. II. Error probabilities Genome Res 1998 8 186 194 9521922

[B41] Gordon D Abajian C Green P Consed: a graphical tool for sequence finishing Genome Res 1998 8 195 202 9521923 10.1101/gr.8.3.195

[B42] Gordon D Desmarais C Green P Automated finishing with autofinish Genome Res 2001 11 614 625 10.1101/gr.171401 11282977 PMC311035

[B43] Bocs S Cruveiller S Vallenet D Nuel G Medigue C AMIGene: Annotation of MIcrobial Genes Nucleic Acids Res 2003 31 3723 3726 10.1093/nar/gkg590 12824403 PMC168996

